# Lack of *TERT* Promoter Mutations in Human B-Cell Non-Hodgkin Lymphoma

**DOI:** 10.3390/genes7110093

**Published:** 2016-10-25

**Authors:** Gary Lam, Rena R. Xian, Yingying Li, Kathleen H. Burns, Karen L. Beemon

**Affiliations:** 1 Department of Biology, Johns Hopkins University, Baltimore, MD 21210, USA; glam5@jhu.edu (G.L.); yli9@jhmi.edu (Y.L.); 2 Department of Pathology, Johns Hopkins Medical Institutes, Baltimore, MD 212105, USA; RXian@mednet.ucla.edu (R.R.X.); kburns@jhmi.edu (K.H.B.); 3 Department of Pathology, University of California Los Angeles, Los Angeles, CA 90095, USA

**Keywords:** Telomerase, non-Hodgkin lymphoma, *TERT* promoter

## Abstract

Non-Hodgkin lymphomas (NHL) are a heterogeneous group of immune cell neoplasms that comprise molecularly distinct lymphoma subtypes. Recent work has identified high frequency promoter point mutations in the telomerase reverse transcriptase (*TERT*) gene of different cancer types, including melanoma, glioma, liver and bladder cancer. *TERT* promoter mutations appear to correlate with increased TERT expression and telomerase activity in these cancers. In contrast, breast, pancreatic, and prostate cancer rarely demonstrate mutations in this region of the gene. *TERT* promoter mutation prevalence in NHL has not been thoroughly tested thus far. We screened 105 B-cell lymphoid malignancies encompassing nine NHL subtypes and acute lymphoblastic leukemia, for *TERT* promoter mutations. Our results suggest that *TERT* promoter mutations are rare or absent in most NHL. Thus, the classical *TERT* promoter mutations may not play a major oncogenic role in TERT expression and telomerase activation in NHL.

## 1. Introduction

Non-Hodgkin lymphomas (NHL) are a heterogeneous group of B, T, and natural killer cell neoplasms that arise primarily in lymph nodes. Most NHL in the western hemisphere are B-cell derived and comprise a variety of lymphomas, with diffuse large B-cell lymphoma (DLBCL), follicular lymphoma (FL), and chronic lymphocytic leukemia/small lymphocytic lymphoma (CLL/SLL) being the most common [1]. Recent advances in molecular genetics have confirmed the molecular heterogeneity of NHL. Classically, NHL can be characterized by chromosomal translocation events that have been shown to occur frequently with different subtypes of NHL [2,3,4]. Whole exome sequencing has further expanded molecular characterizations of NHL. Parallel sequencing experiments with DLBCL patients [5] and FL patients [6] have identified recurrent mutations in functionally relevant genes as well as novel genes that have not been previously implicated. Despite these advances, NHL remains a heterogeneous group of malignancies, with many less characterized subtypes that remain difficult to diagnose and treat with current therapeutic strategies [7].

Recently, non-coding sequences have become an emerging field of active investigation in cancer research [8]. In 2013, specific high frequency promoter mutations in the telomerase reverse transcriptase (*TERT*) gene in melanoma were reported, and were associated with a two- to four-fold increase in transcriptional activity [9,10]. *TERT* encodes the catalytic subunit of telomerase, an enzyme that preserves chromosomal ends through telomere maintenance. The reported somatic transitions −124C>T and −146C>T in the *TERT* promoter region create a novel binding site for the ETS transcription factor GABP, which increases transcription of *TERT* [11]. Increased TERT expression may confer increased proliferative potential and cell survival, which are essential factors in tumorigenesis [12]. Strikingly, *TERT* promoter mutations are not unique to melanomas, but have been later found to be frequent in many other malignancies such as hepatocellular carcinoma, bladder cancer, and glioblastoma [13,14,15,16,17,18,19]. However, *TERT* promoter mutations are not universal. Mutations have been shown to be absent, or rarely observed, in other cancer types like breast, pancreatic, and prostate cancer [13,14,19].

Our lab has used the avian leukosis virus (ALV) as a tool to screen for common proviral integration sites in the host chicken genome to assess events involved in lymphoma development. By high-throughput sequencing and inverse PCR, we have previously shown that early chicken TERT (chTERT) expression through proviral integrations is associated with a similar two- to four-fold increase in transcriptional activity [20,21] and is likely important in lymphomagenesis. Although lymphocytes are known to be a cell type characterized by high telomerase activity throughout their life cycle, lymphoid malignancies are associated with elevated TERT expression like the majority of cancers, suggesting a requirement for persistent TERT activity in transformed cells [22,23].

We sought to investigate whether *TERT* promoter mutations play a role in TERT activation in human lymphomas. Presently, published work on the *TERT* promoter status of NHL is limited. Since the original reports in melanoma, we have found some published work that suggests *TERT* promoter mutations are absent in DLBCL and CLL [15,16]. In contrast, *TERT* promoter mutations were detected in primary central nervous system lymphoma [24]. Here, we report a *TERT* promoter mutation screen of a collection of 105 human B-cell malignancies encompassing nine different subtypes of NHL. Our results indicate that *TERT* promoter mutations are absent across all tested NHL. These findings suggest that *TERT* promoter mutations are not major drivers for TERT up-regulation in lymphomas in contrast to the aforementioned cancers.

## 2. Materials and Methods

### 2.1. Patients and Samples

Representative cases of a variety of B-cell neoplasms were obtained from archived formalin-fixed paraffin-embedded (FFPE) tissues as well as frozen cells and tissues previously banked as de-identified research samples after obtaining institutional review board approval (Johns Hopkins Institution Review Board no. NA_00028682). The FFPE archives were searched from 2000 to 2014 for cases of Burkitt lymphoma, chronic lymphocytic leukemia/small lymphocytic lymphoma, diffuse large B-cell lymphoma, follicular lymphoma, lymphoplasmacytic lymphoma, mantle cell lymphoma, marginal zone lymphoma, myeloma/plasmacytoma, and plasmablastic lymphoma. Cases of glioblastoma and reactive lymph nodes were also queried as expected positive and negative control cases for formalin-fixed paraffin-embedded (FFPE) tissue. Representative cases with unambiguous pathologic diagnoses and sufficient material were selected for histologic re-review by a board-certified Pathologist (Rena R. Xian). Both tumor neoplastic cell content and tissue adequacy was assessed, and only cases with at least 25% neoplastic cells and sufficient tissue were selected for the study. FFPE tissue sections were then acquired from the respective archival cases for subsequent analysis. B-cell acute lymphoblastic leukemia (B-ALL) bone marrow aspirate samples with at least 15% lymphoblasts, and normal bone marrow aspirate samples without phenotypic abnormalities were consecutively collected over a two-month period from remnant material from routine clinical flow cytometric testing. Fresh aspirate material collected in EDTA blood tubes was obtained and frozen until further analysis.

### 2.2. DNA Isolation and Mutational Analysis

DNA extraction from bone marrow was performed using Qiagen DNeasy Blood and Tissue Kit (Valencia, CA, USA) according to manufacturer’s protocol. DNA extraction from FFPE tissue slides was performed using Pinpoint Slide DNA Isolation System^TM^ (Irvine, CA, USA) according to manufacturer’s protocol. Primers with the sequences 5′-M13F-CGGGCTCCCAGTGGATTCGC-3′ and 5′-CGGGGCCGCGGAAAGGAA-3′ were used to PCR-amplify the proximal TERT promoter region containing −124C>T (chr5:1295228, NM_198253, GRCh37/hg19) and −146C>T (chr5:1295250, NM_198253, GRCh37/hg19). Amplified products were then sequenced using standard Sanger sequencing techniques (Louisville, KY, USA) with the universal sequencing priming site, M13F.

## 3. Results

### Absence of TERT Promoter Mutations in NHLs

**Figure 1:**
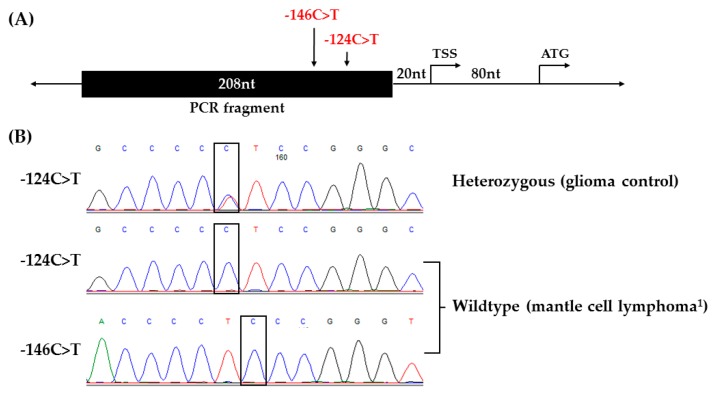
Screening of *TERT* promoter mutations in Non-Hodgkin lymphomas (NHLs). (**A**) Schematic of the amplified region and the location of −124C>T and −146C>T in the *TERT* promoter. (**B**) Sequencing chromatographs of the *TERT* promoter locus in a glioma control that is heterozygous for −124C>T (top) and a representative NHL tumor sample that is wildtype at both positions (middle and bottom). ^1^ A representative trace for wildtype at both positions.

NHLs (Table 1) were screened for *TERT* promoter mutations. The PCR amplified region encompasses the two most commonly mutated nucleotides −124C>T (chr5:1295228G>A) and −146C>T (chr5:1295250G>A) upstream of the translational start site of *TERT* (Figure 1A). Altogether, 93 tumor samples were evaluated with at least 7 samples of each subtype in additional to a subset of gliomas as positive controls, and reactive lymph nodes and normal bone marrows as negative controls (Table 1). We confirmed *TERT* promoter mutations in glioblastomas (n = 2), which is lower than expected [13,15,16] but demonstrates that we can detect the mutation. A glioma control trace that is heterozygous for the −124C>T mutation is shown (Figure 1B). Glioblastoma samples (n = 7) previously identified with promoter mutations were used as additional positive controls. All 7 control samples were confirmed to have the −124C>T mutation. No *TERT* promoter mutations were detected in any NHL samples in the amplified promoter region. A representative NHL trace showing the wildtype *TERT* promoter sequences at both positions are shown from a mantle cell lymphoma tumor sample (Table 1 and Figure 1B). 

## 4. Discussion

As a terminally differentiated cell type, normal human lymphocytes have atypical telomere and telomerase biology. In contrast to other cell types, lymphocytes have above average telomere length and telomerase activity [22,24]. Despite the presence of longer telomeres, and enhanced telomerase activity, lymphocytes still experience division-dependent telomere shortening. Malignant transformation is associated with increased TERT expression and telomere length [25]. Furthermore, longer telomeres and higher telomerase activity are associated with more aggressive NHL than indolent ones, and have been suggested to be a prognostic risk factor for NHL [26]. Our lab has repeatedly observed TERT activation in chicken lymphomas via ALV integration into the *chTERT* promoter region as an early event in avian B-cell lymphomagenesis [20,21].

Taken together, to overcome the restriction of telomere shortening, and support higher proliferative potential and survival, we hypothesized that lymphocytes may acquire *TERT* promoter mutations in the process of malignant transformation, which can directly up-regulate TERT expression and drive telomerase function. However, our data suggest that the NHLs tested were free of the two most prevalent *TERT* promoter mutations [9,10]. This result does not exclude the possibility of promoter mutations further upstream of the area we investigated, and the small sample size does not exclude the presence of low-frequency *TERT* promoter mutations, which would require much larger screens to resolve.

Concurrent to this study, *TERT* promoter mutations were reported to be present in 33% of circulating mantle cell lymphoma [27]. In this study, a limited subset of other lymphoid neoplasms were reported to be free of *TERT* promoter mutations. While we detected no *TERT* promoter mutations in our set of mantle cell lymphoma, there are a few possible reasons for this apparent discordance. First, the small sample size of mantle cell lymphoma (n = 12) evaluated in the current study may be too small to detect a change that may be present in a small fraction of cases. Second, the source of neoplastic B-cells in our study was different from the concurrent study. The peripheral blood source of mantle cell lymphoma in the concurrent study indicates that all patients had circulating leukemic-phase disease, which is associated with advanced stage disease, and worse prognosis when coupled with nodal involvement [28]. This is compared to the node-based disease selected in our study, irrespective of circulating cells, which may harbor different clonal abnormalities commensurate with the stage of the lymphoma. *TERT* promoter mutations may be more prevalent in a particular stage in mantle cell lymphomagenesis. This has been previously shown to be true in melanoma, in which *TERT* mutations are associated with different histology types of the disease, and are more commonly found in melanoma without regression as compared to melanoma with regression [29].

Our findings suggest that activation of TERT expression by acquired *TERT* promoter mutations is not a major driver for TERT activation in NHL. As observed previously, the frequency of this phenomenon is perhaps associated with the intrinsic proliferative potential of the cell type, in which cells with higher proliferative potential like lymphocytes are less likely to have *TERT* promoter mutations [15]. In the case of lymphocytes, perhaps activation of TERT expression indirectly through the up-regulation of other genes like *MYC* is far more common, and supplants the requirement of other mechanisms of TERT activation like *TERT* promoter mutations. Despite our results, the non-coding sequences of NHL tissues remain an uncharted territory, as novel mutated regulatory sites are being discovered across many different cancers [30], and future analysis by whole-genome sequencing may lead to the discovery of novel mechanisms in lymphomagenesis. As whole-genome patient sequencing and clinical data becomes available, we can begin to explain these observations and apply them to enhance our understanding of cancer biology and the treatment of cancer.

## 5. Conclusions

Our results support the view that *TERT* promoter mutations are rare or absent in most NHL, and likely not major drivers of TERT activation.

## Figures and Tables

**Table 1:** Samples tested for telomerase reverse transcriptase (*TERT*) promoter mutations ^1^.

Tumor Type	No. of Tumors	No. of Tumors Mutated
B-cell acute lymphoblastic leukemia	12	0
Burkitt lymphoma	9	0
Chronic lymphocytic leukemia	11	0
Diffuse large B-cell lymphoma	9	0
Follicular lymphoma	13	0
Lymphoplasmacytic lymphoma	7	0
Mantle cell lymphoma	12	0
Marginal zone lymphoma	16	0
Myeloma/plasmacytoma	9	0
Plasmablastic lymphoma	7	0

^1^ Glioblastoma tissues were used as positive controls (n = 11); reactive lymph nodes (n = 13) were used as negative controls for formalin-fixed paraffin-embedded (FFPE) samples; normal bone marrow samples (n = 13) were used as negative controls for B-cell acute lymphoblastic leukemia (B-ALL) samples.
